# Supplementary material for: Complexome profiling of the Chlamydomonas psb28 mutant reveals TEF5 as an early PSII assembly factor
Source: Plant Cell. 2025 Mar 17;37(6):koaf055. doi: 10.1093/plcell/koaf055 (PMC12164590; doi:10.1093/plcell/koaf055)
Supplement: koaf055_Supplementary_Data [file koaf055_supplementary_data.zip › psb28_interactive.zip!/plots/intensity/qualityPlots/peptides/FAD3_Cre01.g037700.t1.2.html]

### Cre01.g037700.t1.2

Trivial name: FAD3  
SeqCoverage [median]: 21.10  
Mass: 56.88 kDa  
  
 

| Sequence | MissedCleavage | Mass [Da] | wtIntensity | muIntensity | Score |
| --- | --- | --- | --- | --- | --- |
| DATALFESYHLRPEVAASMLK | 1 | 2348.18 | 621.70 | 5248.12 | 92.26 |
| DGEMLMLSK | 0 | 1022.48 | 0.00 | 394.14 | 57.59 |
| ENLDESAVTR | 0 | 1132.54 | 5979.52 | 13883.89 | 200.68 |
| EVFKGTEIK | 1 | 1049.58 | 0.00 | 1674.13 | 42.40 |
| EVGAAPQKPVK | 1 | 1122.64 | 0.00 | 1020.52 | 60.52 |
| EVGAAPQKPVKR | 2 | 1278.74 | 301.67 | 525.66 | 73.50 |
| GIPYSHYDTLPEILGR | 0 | 1829.93 | 1281.43 | 0.00 | 43.81 |
| GVAYDLTPYLDRHPGGR | 1 | 1885.94 | 1096.77 | 0.00 | 43.80 |
| TVGATLYGASNFER | 0 | 1484.73 | 2894.13 | 15232.57 | 149.31 |
|  |  |  | 12175.2 | 37979.0 |  |
